# Supplementary material for: Patterns of Tadpole β Diversity in Temperate Montane Streams
Source: Animals (Basel). 2024 Apr 20;14(8):1240. doi: 10.3390/ani14081240 (PMC11047721; doi:10.3390/ani14081240)
Supplement: Supplementary file 1 [file animals-14-01240-s001.zip › animals-2927211-supplementary.pdf]

Table S1. Geographical information on the 18 selected transects in Mount Emei.

Name	Code	Longitude (E)	Latitude (N)	Elevation (m)
Huangwanfangqu	YX1	103.4367	29.5824	485
Huangwan	YX2	103.4377	29.5874	509
Baoguosi	YX3	103.4408	29.5689	563
Shenshuigedahe	YX4	103.4039	29.5607	638
Lianghekou	YX5	103.4106	29.5866	658
Qingyinge	YX6	103.3944	29.5717	708
Shenshuige	YX7	103.4088	29.5659	807
Chadicunxiagou	YX8	103.3598	29.5965	928
Heishuicun	YX9	103.3610	29.6021	950
Chadicunshanggou	YX10	103.3181	29.5983	1113
Longdonghe	YX11	103.2827	29.5820	1242
Longdonghezhiliu	YX12	103.2816	29.5785	1248
Linggonglizhugou	YX13	103.2916	29.5855	1320
Linggonglizhigou	YX14	103.2908	29.5864	1323
Changshouqiao	YX15	103.3505	29.5594	1560
Longqiaogou	YX16	103.3523	29.5522	1602
Shuangshuijin	YX17	103.3209	29.5471	2243
Jinding	YX18	103.3332	29.5237	2865

Table S2. The averages and standard deviation values of microhabitat variables.

Microhabitat variables	Average	Standard deviation
water temperature (℃)	15.20	± 2.77
water pH	7.99	± 0.32
water conductivity (μs/cm)	263.83	± 141.86
dissolved oxygen (μmol/L)	8.24	± 0.54
current velocity (m/s)	0.26	± 0.15
water depth (cm)	27.89	± 8.81
river width (m)	4.20	± 2.57
total phosphorus (mg/L)	0.02	± 0.01
total nitrogen (mg/L)	3.00	± 0.99
ammonium nitrogen (mg/L)	0.17	± 0.06
chlorophyll α(mg/L)	0.34	± 0.53

Table S3. Species distribution (relative number of individuals, %) in each transect. Details of abbreviations are provided in Table S1.

Scientific name	YX1	YX2	YX3	YX4	YX5	YX6	YX7	YX8	YX9	YX10	YX11	YX12	YX13	YX14	YX15	YX16	YX17	YX18
Bufo gargarizans	3.25	0	0	0	0	0	0	6.54	0	47.58	70.51	43.40	0	0	0	0	0	0
Fejervarya multistriata	46.77	0	0	0	0	0	0	0	0	0	0	0	0	0	0	0	0	0
Quasipaa boulengeri	0	2.08	0	4.00	0.64	0.56	42.86	3.92	0	0.85	0	0	0.75	0	0	0	0	0
Leptobrachium boringii	0	97.92	63.16	52.00	60.90	43.50	10.71	6.54	0	5.98	0	0	0	0	0	0	0	0
Megophrys minor	0	0	2.63	0	0	1.69	17.86	0	0	0	0	0	3.01	6.56	1.64	0	0	0
Megophrys omeimontis	0	0	0	4.00	23.08	35.03	0	47.06	0	18.23	0	0	29.32	32.79	22.95	0	0	0
Megophrys shapingensis	0	0	0	0	0	0	0	0	0	0	0	0	0	0	0	2.63	0	0
Oreolalax major	0	0	0	0	0	0	0	0	0	0	0	0	0	0	0	2.63	0	0
Oreolalax omeimontis	0	0	2.63	8.00	0	4.52	3.57	20.26	0	25.07	0	9.43	66.92	60.66	62.30	86.84	0	0
Oreolalax popei	0	0	0	0	0	1.69	0	0	0	0.28	0	0	0	0	13.11	5.26	0	0
Oreolalax schmidti	0	0	0	0	0	0	0	0	0	0	0	0	0	0	0	2.63	0	0
Paramegophrys oshanensis	0	0	0	0	15.38	0.56	21.43	0	0	0	0	0	0	0	0	0	0	0
Microhyla fissipes	3.23	0	0	0	0	0	0	0	0	0	0	0	0	0	0	0	0	0
Amolops chunganensis	0	0	0	0	0	1.13	0	0	0	0	0	0	0	0	0	0	0	0
Amolops granulosus	0	0	0	4.00	0	0	0	1.96	0	0	0	0	0	0	0	0	0	0

Scientific name	YX1	YX2	YX3	YX4	YX5	YX6	YX7	YX8	YX9	YX10	YX11	YX12	YX13	YX14	YX15	YX16	YX17	YX18
Odorrana margaretae	0	0	31.58	24.00	0	11.30	0	6.54	0	0.28	0	0	0	0	0	0	0	0
Pelophylax nigromaculatus	46.77	0	0	4.00	0	0	0	0	0	0	0	0	0	0	0	0	0	0
Rana omeimontis	0	0	0	0	0	0	0	0	0	0	29.49	37.34	0	0	0	0	0	0
Rhacophorus omeimontis	0	0	0	0	0	0	3.57	7.19	0	0	0	9.43	0	0	0	0	0	0

Table S4. Relationships between distance of predictor variables (i.e., geographical, elevational and environmental distance) and multifaceted β diversity based on liner regression models.

β diversity components	Predictors	Intercept	Slope	r	*p*
Total dissimilarity	Geographical distance	0.527	0.017	0.279	**0.007**
Turnover		0.357	0.022	0.267	**0.008**
Nestedness		0.170	-0.005315	-0.153	0.952
Total dissimilarity	Elevational distance	5.442e-01	2.342e-04	0.273	**0.010**
Turnover		0.367	0.0003	0.291	**0.008**
Nestedness		1.774e-01	-9.990e-05	-0.176	0.962
Total dissimilarity	Environmental distance	0.222	0.091	0.465	**0.006**
Turnover		-0.039	0.118	0.440	**0.007**
Nestedness		0.262	-0.027	-0.244	0.959

The bold values indicate $p < 0.05$.

Table S5. Independent contribution of each explanatory distance matrix in the variations of multifaceted β diversity. The meaning of the abbreviations can be found in Table 2.

	Total dissimilarity	Turnover	Nestedness
Geo	14.77%	12.62%	4.58%
Ele	5.75%	9.07%	13.73%
pH	0.12%	0.25%	0.51%
Con	1.22%	4.33%	14.22%
Do	0.38%	0.79%	1.70%
Rw	27.26%	21.60%	5.64%
Wd	0.28%	0.94%	16.41%
Cv	10.03%	15.47%	22.66%
chl.a	33.62%	27.68%	8.62%
TN	2.90%	5.25%	9.52%
NN	0.59%	0.56%	0.30%
TP	3.06%	1.33%	0.17%
Sub	0.03%	0.12%	1.94%
